# Outcomes of treatment of severe COVID-19 pneumonia with tocilizumab: a report of two cases from Tunisia

**DOI:** 10.11604/pamj.2021.40.126.28020

**Published:** 2021-11-02

**Authors:** Mouna Ben Azaiz, Bassem Chatbri, Walid Sellami, Chihebeddine Romdhani, Khaled Lamine, Ezzedine Ghazouani, Ridha Oueslati, Hedi Gharsallah, Mustapha Ferjani

**Affiliations:** 1 Laboratory of Immunology, Military Hospital of Tunis, Montfleury - 1008, Tunis, Tunisia,; 2 Faculty of Medicine, University Tunis El Manar, Tunis, Tunisia,; 3 Research Unit 17 DN05, Military Hospital of Tunis, Montfleury - 1008, Tunis, Tunisia,; 4 Emergency Department, Military Hospital of Tunis, Montfleury - 1008, Tunis, Tunisia,; 5 Intensive Care Unit, Military Hospital of Tunis, Montfleury - 1008, Tunis, Tunisia,; 6 Laboratory of Immunology, Faculty of Science, Bizert, Tunis, Tunisia

**Keywords:** COVID-19 infection, cytokine storm, tocilizumab, interleukin 6, case report

## Abstract

The SARS CoV-2 pandemic is a global health threat with high morbidity and mortality (1 to 4%) rates. COVID-19 is correlated with important immune disorders, including a “cytokine storm”. A new therapeutic approach using the immunomodulatory drug, Anti-IL6 (tocilizimub), has been proposed to regulate it. We report here the first Tunisian experience using tocilizimub in two severe cases of COVID-19 pneumonia. The diagnosis was confirmed by chest scan tomography. Biological parameters showed a high level of Interleukin-6 (IL-6) that increased significantly during hospitalization. The patients developed hypoxia, so they received intravenously 8 mg/kg body weight tocilizumab. There was a resultant decrease in the level of IL6, with clinically good evolution. Blocking the cytokine IL-6 axis is a promising therapy for patients developing COVID-19 pathology.

## Introduction

The SARS CoV-2 pandemic is a global health threat with high morbidity and mortality (1 to 4%) rates [1]. There is still significant work required to understand the pathogenesis of this disease, and we need to discover effective medication that could be used for treatment. The severe forms of COVID-19 are frequently correlated with immune disorders, including lymphopenia and a very intense cytokine immune response which has been qualified as a “cytokine storm”, mainly involving interleukin 6 (IL-6) [2, 3].

Based on clinical and biological data collected from patients infected with SARS CoV-2, tocilizumab was used in several clinical trials aiming to regulate the “cytokine storm” [4]. Tocilizumab is a recombinant humanized anti-human IL-6 receptor monoclonal antibody that specifically binds soluble IL-6 receptor and inhibits signal transduction [5]. Considering the guidelines (7^th^ edition) published by the National Health Commission of China, tocilizumab was used in our severe COVID-19 patients [6]. Severe cases are defined as having at least one of the four criteria: (i) a respiratory rate of at least 30 breaths per minute; (ii) peripheral blood oxygen saturation (SaO_2_) lower than 93% in room air; (iii) a ratio of arterial oxygen partial pressure (PaO_2_) to fractional inspired oxygen (FiO_2_) lower than 300 mmHg in room air; and (iv) infiltrates affecting more than 50% of the lungs´ volume within 24-48h of admission to the hospital. We report herein the outcomes of treatment with tocilizumab in two patients with severe COVID-19.

## Patient and observation

### Case 1

**Patient information´s:** an 83-year-old male, with a history of thyroid cancer, chronic obstructive pulmonary disease (COPD) and an ischaemic stroke, was admitted to the emergency unit of the Military Hospital of Tunis (Tunisia) for a dry cough and dyspnoea.

**Clinical findings:** when examined, he had blood pressure 120/70mmH; pulse 85/min; respiratory rate 28/min and a spot oxygen saturation (SpO_2_) 78%. He developed a fever (38.3°C) prompting a SARS-Cov-2 real-time polymerase chain reaction (qPCR) test, which was negative from a nasopharyngeal swab but positive from sputum.

**Figure 1:**
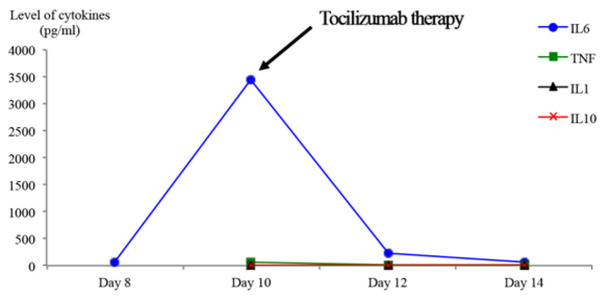
the levels of inflammatory and anti-inflammatory cytokines during COVID-19 infection and after the use of Tocilizumab therapyfor patient 1 (day 0: is the day admission tohospital)

**Diagnostic assessment:** chest computerized tomography (CT) scan revealed bilateral patchy ground-glass opacities related to COVID-19. Biological parameters at admission showed a high level of C-reactive protein (CRP) at 224 mg/L(8 mg/ml threshold) and a lymphopenia at 900 cells/mm^3^ (normal range: 1000-7000 cells/mm^3^) and a high blood concentration level of Interleukin-6 (IL-6) at 75.6pg/ml (threshold 5 pg/ml) (Figure 1).

**Therapeutic Intervention:** the patient was under oxygen therapy (5l/mn). He was treated with an association of hydroxychloroquine (400 mg per day), ceftriaxone (2g per day), azithromycin (500mg per day), and curative anticoagulation with low-molecular-weight-heparin dose subcutaneously once a day. Hydroxychloroquine was withdrawn after 48 hours because of the occurrence of arrhythmia.

**Timeline:** day 6, the patient developed hypoxia, requiring high oxygen flow administration without mechanical ventilation. The Sequential Organ Failure Assessment (SOFA) score of the patient was evaluated at 2, mainly driven by respiratory failure and requirement of high oxygen support. The IL-6 concentration increased significantly to 3432pg/ml (Figure 1). Day 10, the patient received intravenously 8 mg/kg body weight tocilizumab.

**Follow-up and outcomes:** day 12, the status of the patient improved with a partial regression of the pulmonary infiltrates and ground glass appearance as shown by the pulmonary CT. There was a decrease in the level of IL-6 (227 pg/ml) (Figure 1). In day 13, CRP level (0 mg/ml) and lymphocytes (2020 cells /mm^3^) were normal and the patient was discharged at day 20.

### Case 2

**Patient information´s:** a 47-year-old woman, with a history of dyslipidaemia.

**Clinical findings:** she was admitted to the emergency unit of the Military Hospital of Tunis (Tunisia) for a dry cough, dyspnea, and fever. The clinical examination showed that haemodynamic indicators were stable with no signs of hypoxia and a negative SARS-Cov-2 nasopharyngeal swab qPCR.

**Figure 2:**
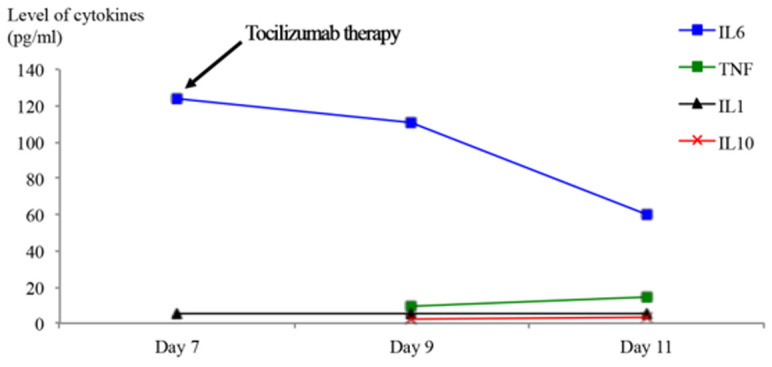
the levels of inflammatory and anti-inflammatory cytokines during COVID-19 infection and after the use of Tocilizumab therapy for patient 2 (day 0: is the day admission to hospital)

**Diagnostic assessment:** chest computerized tomography (CT) scan revealed bilateral patchy ground-glass opacities related to COVID-19. There was a high level of CRP (51 mg/l) with normal blood lymphocyte concentration (2450/mm^3^) and the level of IL-6 was123 pg/ml (Figure 2).

**Therapeutic Intervention:** the patient was treated with hydroxychloroquine (400 mg per day), ceftriaxone (2g per day), azithromycin (500mg per day), and a subcutaneous injection of low molecular weight heparin according to the local policy at that time.

**Timeline:** day 5, the patient developed hypoxia, requiring high oxygen flow administration without mechanical ventilation. The Sequential Organ Failure Assessment (SOFA) score was 2, mainly driven by respiratory failure and requirement of high oxygen support. Day 7, the patient received a perfusion of tocilizumab (8 mg/kg bodyweight) over one hour.

**Follow-up and outcomes:** day 11, chest CT showed partial regression of pulmonary infiltrates and ground glass appearance. Day 13, the patient was discharged. There was a decrease in the level of IL-6 (60 pg/ml) (Figure 2).

**Patient perspective:** the patients were unable to talk, but their parents have accepted the treatment with great hope of success.

**Informed consent:** the parents of patients was informed about the treatment.

## Discussion

Inflammatory cytokine storm is very common in patients with COVID-19 [2, 3]. A meta-analysis showed a positive correlation between pro-inflammatory cytokine levels (IL-6, IL-10, IL-2R, IL-8 and CRP) and severity and mortality in SARS-Cov-2 infected patients [7]. This new finding in the pathophysiology of COVID-19 and in particular the cytokine profile led to a new therapeutic approach: the administration of immunomodulatory compounds in order to regulate the “cytokine storm” and first results were encouraging [4, 8].

We selected two patients with severe COVID-19 but with no organ failure and high level of IL-6 without clinical signs of immunodeficiency or lymphopenia. Very high level of IL-6 were found in the two patients prior to tocilizumab administration (maximum: 3400 pg/ml). We injected, as recommended, intravenous injection of tocilizumab in a single dose of 400 mg/patient [4, 8]. It gave clinical and biological good results. In the two patients, the IL-6 decreased and lymphocytes count returned to normal level and did not develop infections. Although the effectiveness of some other molecules is controversial, a phase III trial of sarilumab (Kevzara®) at 400 mg in COVID-9 patients requiring mechanical ventilation did not meet its primary and key secondary endpoints when sarilumab was added to the best supportive care compared, supportive care alone and a placebo [9].

These differences could be explained by the timing of administration, before or after multi-systemic failure. The success of the protocol that was adopted herein could be explained by the close monitoring that allowed treatment of the patients at the right time. A specific phenotype of patients without organ failure seems to be a key element for the efficacy of tocilizumab [10, 11]. Huang *et al*. [12] reported in patients who developed a severe form of Covid-19 infection an increase of IL-10, an anti-inflammatory cytokine, and normal values of IL-6. In this case, it may be dangerous to accentuate the anti-inflammatory condition using tocilizumab. The current justification for inhibiting IL-6 is also controversial, as IL-6 promotes antibody synthesis [13].

Even with some limits, using an anti IL6 drug is a challenging new approach for treating COVID-19 patients that needs to be considered, mainly in severe forms of this disease in addition to local standard of care therapy. Since pro-inflammatory IL-6 axis seems to play a major role in “cytokine storm” development, close monitoring of biological parameters, cytokine and lymphocyte profiles may be useful to reduce any risk of infectious complications occurring.

## Conclusion

In these two severe COVID-19 cases, we successfully used, for the first time in Tunisia, tocilizumab in association with the local standard therapeutic protocol and close monitoring of IL-6. Thus, blocking the cytokine IL-6 axis appears to be a promising therapy for COVID-19 patients. There is still significant work required to understand the pathogenesis of this disease and, mainly the “cytokine storm”, for better management of severe COVID-19 cases.
